# Patient-centred outcomes of imaging tests: recommendations for patients, clinicians and researchers

**DOI:** 10.1136/bmjqs-2021-013311

**Published:** 2021-10-06

**Authors:** Matthew J Thompson, Monica Zigman Suchsland, Victoria Hardy, Danielle C Lavallee, Sally Lord, Emily Beth Devine, Jeffrey G Jarvik, Steven Findlay, Thomas A Trikalinos, Fiona M Walter, Roger Chou, Beverly B Green, Karen J Wernli, Annette L Fitzpatrick, Patrick M Bossuyt

**Affiliations:** 1 Department of Family Medicine, University of Washington, Seattle, Washington, USA; 2 Department of Public Health and Primary Care, University of Cambridge, Cambridge, UK; 3 Department of Health Systems and Population Research, University of Washington, Seattle, Washington, USA; 4 The University of Sydney, Sydney, New South Wales, Australia; 5 The Comparative Health Outcomes, Policy and Economics (CHOICE) Institute, School of Pharmacy, University of Washington School of Pharmacy, Seattle, Washington, USA; 6 Departments of Radiology and Neurological Surgery, University of Washington, Seattle, Washington, USA; 7 Barnesville, Maryland, USA; 8 Departments of Health Services, Policy & Practice, and Biostatistics, Brown University School of Public Health, Providence, Rhode Island, USA; 9 Wolfson Institute of Population Health Science, Barts and The London School of Medicine and Dentistry, Queen Mary University of London, London, UK; 10 Department of Medical Informatics & Clinical Epidemiology, Oregon Health & Science University, Portland, Oregon, USA; 11 Kaiser Permanente Washington Health Research Institute, Seattle, Washington, USA; 12 Departments of Family Medicine, Epidemiology, and Global Health, University of Washington, Seattle, Washington, USA; 13 Epidemiology & Data Science, Amsterdam Public Health, Amsterdam University Medical Centres, Amsterdam, The Netherlands

**Keywords:** comparative effectiveness research, decision support, clinical, evaluation methodology, patient-centred care, quality improvement methodologies

## Abstract

**Background:**

Imaging tests are one of the most frequently used diagnostic modalities in healthcare, but the benefits of their direct impacts on clinical decision-making have been countered by concerns that they can be overused. Assessing the relative value of imaging tests has largely focused on measures of test accuracy, which overlooks more comprehensive benefits and risks of imaging tests, particularly their impact on patient-centred outcomes (PCOs). We present the findings of the Patient Reported Outcomes of Diagnostics (PROD) research study in response to a methodological gap in the area of diagnostic test comparative effectiveness research.

**Methods:**

Over a 3-year period, the PROD Study engaged with multiple stakeholders to identify existing conceptual models related to PCOs for imaging testing, conducted primary research and evidence synthesis, and developed consensus recommendations to describe and categorise PCOs related to imaging testing.

**Results:**

The PROD framework categorises PCOs from imaging studies within four main domains: information or knowledge yielded, physical impact, emotional outcomes and test burden. PCOs interact with each other and influence effects across domains, and can be modified by factors related to the patient, clinical situation, healthcare team and the testing environment.

**Conclusions:**

Using PCOs to inform healthcare decision-making will require ways of collating and presenting information on PCOs in ways that can inform patient–provider decision-making, and developing methods to determine the relative importance of outcomes (including test accuracy) to one another.

## Introduction

Multiple frameworks have been developed to evaluate diagnostic tests, which typically include generating evidence across phases of technical efficacy, test accuracy, diagnostic efficacy, therapeutic efficacy, patient outcome and societal aspects.^1–4^ While test accuracy plays a pivotal role for clinical outcomes and regulatory approval, there have been repeated demands for more comprehensive methods to evaluate the benefits and risks of diagnostic tests in terms of patient-centred outcomes (PCOs).^5^

The concept that tests can impact patient well-being, in addition to ‘clinical’ outcomes,^6^ has mainly been explored in the context of impacts of false positive screening test results.^7–9^ There have been few attempts to systematically determine which PCOs are important to patients undergoing testing, nor the extent to which these outcomes are shared across different types of tests, and how these outcomes can be used as part of shared decision-making. Patient-centred care is based on the understanding that PCOs include topics that patients themselves identify as important,^10^ which in turn can be used to drive service improvements by comparing performance on outcome metrics that matter to patients.^11 12^ While this concept has been applied to comparative effectiveness research of interventions, it has rarely been applied to diagnostic tests.^5^

The Patient Reported Outcomes of Diagnostics (PROD) Study^13^ aimed to develop consensus-based recommendations to guide methods for incorporating PCOs within comparative effectiveness research of diagnostic tests. We focused on imaging testing, given that this is one of the most frequently used modalities of testing in healthcare, yet faces concerns of overuse and rising costs.^9 14 15^ We used primary research, evidence syntheses and input from multiple stakeholders to describe and categorise PCOs from imaging tests with a goal of informing clinical care, research and policy.

## Methods

### Overview of approach

**Figure 1:**
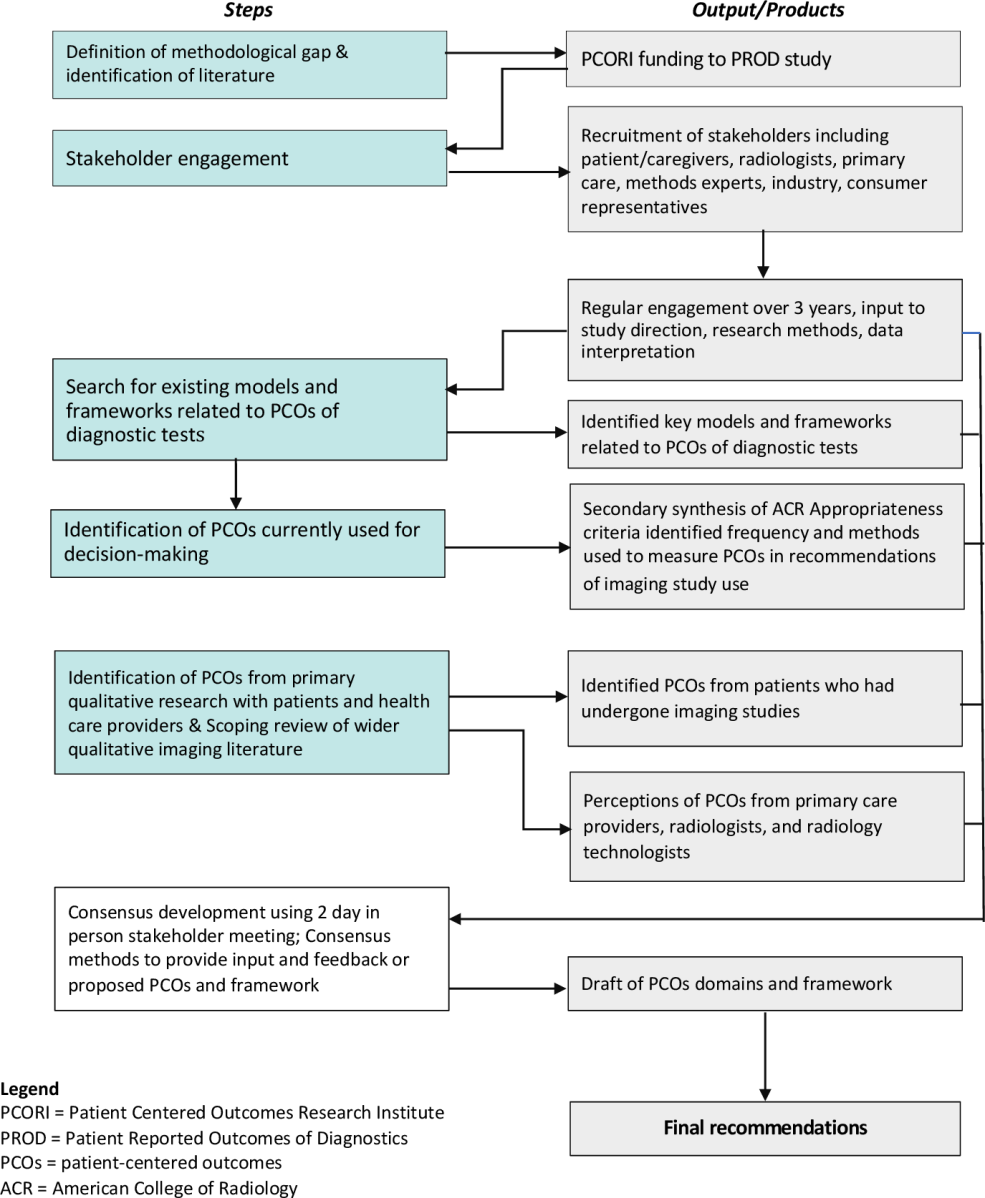
Data sources and methods used to develop final recommendations. Note: This figure was created by the authors and no persmission is required.

Our approach was based on the multistep processes used to develop consensus methods and research reporting guidelines.^12 16–18^ Over a 3-year period, we: (1) confirmed the methodology gap and identified relevant conceptual models and frameworks, (2) recruited and engaged stakeholders, (3) identified PCOs currently used in clinical recommendations for imaging testing, (4) conducted qualitative research with patients and healthcare providers to identify PCOs, (5) conducted a scoping review of PCOs from existing qualitative literature, and (6) developed consensus-based recommendations on PCOs of imaging testing (figure 1).^19^ We considered that consensus methods provided two advantages over other methods (such as Delphi or nominal group technique): first, allowing synthesis of the best available information; and second, allowing a process of consensus and validation between key stakeholders.^20^

#### Confirmation of methodology gap and identification of relevant conceptual models and frameworks

We used an iterative process to identify published literature that had attempted to address the methodological gap, including an extensive search of existing research on diagnostic test evaluation and imaging tests specifically. This was used to provide additional justification for the proposed research and identify any additional evaluation frameworks for diagnostic tests, conceptual models outlining the range of PCOs that may occur with testing, and literature specific to PCOs from imaging tests. We used a non-systematic scan of the literature search, including existing systematic reviews,^1–8^ input from methods experts and focused hand-searching.

#### Stakeholder recruitment and engagement

We adopted the Six-Stage Model for Patient-Centered Outcomes Research and Comparative Effectiveness Research to guide stakeholder engagement.^21^ Stakeholders were selected from the following: (1) patients and patient advocates with support from a patient advisory network (http://becertain.org/partnerships/patient-advisory-network); (2) primary care clinicians and radiology staff from the Washington, Wyoming, Alaska, Montana and Idaho Practice and Research Network; (3) the American College of Radiology (ACR); (4) consumer advocates; (5) imaging industry and (6) diagnostic methods experts. Recruitment was based on a combination of outreach through national, regional and local organisations. The stakeholders guided research direction, informed data collection instruments, interpreted findings and contributed to research outputs.^22 23^ Additionally, stakeholders attended a 2-day meeting to develop the final recommendations.

#### Identification of PCOs currently used in clinical recommendations

In order to identify the frequency and type of PCOs reported in studies of imaging testing that are used to inform clinical recommendations, we conducted a secondary analysis of studies included in the ACR’s Diagnostic Imaging Appropriateness Criteria which are used by referring physicians and radiologists to guide imaging test decisions and are incorporated into clinical decision support mechanisms. We used a broad definition of PCOs used by Patient Centered Outcomes Research Institute (PCORI) and modified by other researchers in this field.^5 6 24^ We searched for PCOs reported in articles published across all clinical areas relevant to the PROD Study (ie, excluding paediatric and obstetric imaging, interventional radiology), and used systematic methods to extract and synthesise data.^25 26^

#### Qualitative research with patients and healthcare providers to identify PCOs

We conducted semistructured interviews with 45 patients who had undergone imaging studies across a variety of conditions and imaging modalities, 16 primary care providers, and 16 radiologists and radiology technologists to seek their experiences and perceptions of PCOs.^25 27^ Patients and clinicians were recruited from primary care clinical sites and radiology offices affiliated with the Washington, Wyoming, Alaska and Montana Practice and Research Network (WPRN). This research also aimed to identify factors that could influence the perceived importance of these outcomes for patients.^28^

#### Scoping review of PCOs from existing qualitative literature

A scoping review of qualitative studies reporting PCOs from imaging studies was used to broaden the evidence base of PCOs beyond those identified from our primary research. We searched for studies reporting PCOs across multiple imaging modalities and clinical settings. The review aimed to identify relevant studies that had explored patients’ emotions, knowledge, and physical preferences in relation to imaging tests either before, during, or after imaging testing. The scoping review, described in full in online supplemental appendix 1, identified and synthesised qualitative research that had reported PCOs from any type of imaging modality, clinical setting and patient group.^29–31^

10.1136/bmjqs-2021-013311.supp1 Supplementary data

#### Development of consensus-based recommendation on PCOs for imaging testing

At the end of the 3-year period, 28 stakeholders (online supplemental appendix 2) participated in a 2-day meeting held in Seattle, Washington, which aimed to define and categorise PCOs related to imaging testing, and provide recommendations for next steps needed to implement PCOs in decision-making. We followed the five key elements of consensus methods as outlined by Black *et al.* 32 (1) Approach to the task: the open-ended study goals were chosen to avoid influencing judgement or selectivity of the stakeholders, and our process included research evidence, experience of consumers (in this case, patients/caregivers) and clinical expertise (in this case, the primary care, radiologists and other stakeholders).^33^ (2) Participant selection: one of the most important components of our consensus method was engaging multiple stakeholders, as described above, representing different potential viewpoints and expertise on PCOs. We considered this would provide a range of values, beliefs and experiences.^20^ (3) Presentation of scientific data: we used the regular stakeholder meetings to engage stakeholders across the entire lifespan of this research. This included direct involvement with developing the primary research studies, input to findings from the primary research (including as coauthors), and presenting emerging descriptions and details of PCOs as they emerged. (4) Structure of the interaction: stakeholder meetings were facilitated by research staff with experience in patient engagement and occurred approximately quarterly teleconference for the initial study period of 2 ½ years. We held additional meetings only attended by patients and caregivers, with the same frequency over this period, to allow their voices to be fully heard. (5) Method of synthesising data: we aimed to achieve conclusions regarding the definition and categorisation of PCOs using a reflexive and iterative process.^34^ Over the initial 2 ½ years of the research period, we synthesised findings from the emerging research at quarterly stakeholder meetings, developing and publishing in peer-reviewed literature the emerging findings. The 2-day in-person conference aimed to debate and review proposed final definitions and categorisation of PCOs, and provide recommendations for next steps needed to implement PCOs in decision-making. We did not intend to use this meeting to eliminate or rank importance of PCOs. Prior to the meeting, stakeholders received preparatory material including descriptions of PCOs that had emerged during the previous 2 ½ years, and lay summaries of additional publications. During the meeting, the research team presented the draft materials and used small group breakout sessions, to seek input on both the clarity of these definitions and whether any PCOs had been overlooked or missed out. We also used small groups to attempt to identify the best way to categorise the PCOs. Following the meeting, multiple written drafts of the consensus recommendations were distributed to stakeholders, and agreement was reached on the final document from all stakeholders.

### Role of funding source

The work was funded by the PCORI, which approved the research plan submitted by the research team, but had no input to the research methods, findings, development of consensus recommendations, nor in preparation or approval for any manuscripts submitted for publication.

### Human subjects approval

All primary qualitative studies conducted by the authors, which are referenced in this manuscript, were approved by the University of Washington Division of Human Subject as documented in those publications.^25 27 28^ The activities of this consensus manuscript (including scoping review and systematic review) were determined to not involve human subjects and did not require additional IRB approval.

## Results

### Confirmation of the methodology gap and identification of relevant conceptual models and frameworks

We identified several recommendations from groups such as guideline development organisations from the USA and Europe, and the Grading of Recommentations Assessment, Development and Evaluation (GRADE) Working Group that supported a need for methods to measure the effects of tests on PCOs.^5 18 35^ The need for methods to broaden the evaluation of imaging testing was identified in several publications, including limitations in reporting of imaging test research.^9 36^ Several studies had suggested a range of potential PCOs from diagnostic tests, but none had defined these in a systematic way.^6–9 37^ This step of the process therefore confirmed that the methods gap that PCORI had identified had not been addressed in other publications.

### Identification of PCOs currently used in clinical recommendations for imaging testing

The secondary analysis of the ACR Appropriateness Criteria identified 89 eligible studies; these covered a wide range of clinical areas and imaging modalities.^26^ The most frequent PCOs identified were: concerns about radiation exposure (n=37), the need for additional testing following an initial test (n=20), test complications (n=19), and indeterminate or incidental findings (n=10). Other PCOs included quality of life (n=7), physical discomfort (n=5), patient values and experiences (n=4), patient financial and time costs (n=4), psychosocial outcomes (eg, depression, anxiety, claustrophobia) (n=4) and test duration (n=2) This analysis highlighted that relatively few PCOs are included in studies that underpin this set of clinical recommendations. The outcomes identified were mainly related to immediate or short-term health complications from the test process itself, and rarely reported from patients themselves.

### Qualitative research with patients and healthcare providers to identify PCOs

Analysis of interviews with 45 patients, 16 primary care providers, and 16 radiologists and radiology technologists identified four themes related to PCOs.^25 27 38^ These were: (1) information or knowledge gained from the test to address patients’ questions and to facilitate next steps in their healthcare; (2) physical experiences during the test procedure, such as discomfort or potential adverse effects; (3) positive and negative impacts of the testing process on patients’ emotions; and (4) the direct and indirect financial burden of testing. This research also highlighted factors that might influence outcomes, such as the effectiveness and content of patient–provider communication, impact of radiology staff, and patients’ previous testing experience, underlying health, level of knowledge, expectations of the imaging test, insurance status, and cultural background.

### Scoping review of PCOs from existing qualitative literature

We identified 25 qualitative studies that described PCOs, mainly focusing on mammography and MRI scanning, and most related to cancer screening, conducted in multiple countries (online supplemental appendix 1). We identified PCOs in three main domains, namely: (1) knowledge or information yielded by the imaging test including the desire to know what is wrong, irrespective of the finding, and a desire to know what individuals might experience both during test preparation and the procedure itself; (2) the emotional impact of the test both during preparatory stages and during the test, and the impact of compassion and empathy from radiology staff; and (3) physical discomfort associated with the testing procedure.

### Development of consensus-based recommendation on PCOs of imaging testing

The stakeholder meeting facilitated discussion and feedback on proposed PCOs, domains and recommendations. The PROD team developed a matrix which proposed PCOs occurring across a range of domains, before, during and after an imaging test. This matrix was shared with stakeholders to develop consensus regarding the full scope of potential PCOs and categorisation into potential domains. The main outcomes, described below, categorised PCOs within four domains, outlined how PCOs can potentially interact with each other and identified factors that can modify how they are experienced.

### Domain 1: information or knowledge yielded by an imaging test

PCOs in this domain included test information that contributes to determining an underlying cause for patients’ symptoms or concerns, or information that led to reducing the likelihood of a condition (table 1). In addition, patients described the value of knowing or seeing these results, regardless to some extent of what the imaging had revealed. Additional PCOs related to the impact of test results on decision-making, such as facilitating access to a higher level of care or a particular treatment course. There were also several negative outcomes, including: misleading information, particularly false positive tests prompting the need for additional testing to confirm/rule out a condition; inconclusive or indeterminate results that did not provide a definitive diagnosis and could lead to further testing; and unexpected or incidental findings that might or might not have clinical significance, but require additional testing or investigations with associated burdens and impact on PCOs.

**Table 1:** Patient-centred outcomes within domain of information or knowledge yielded by imaging tests

Outcomes	Definition/explanation
Finding cause of symptoms	Finding out what is causing symptoms With a known diagnosis, information that leads to finding out how serious it is The desire for a definitive diagnosis, to reduce uncertainty
Reducing the probability of a condition that patient worried about	Excluding a serious condition based on the test results
Value of just knowing or finding out more, whatever the outcome	The desire to know, to find answers or just to find out/see something in their own body
Decision-making around the information given by the test, leading to action	Test results facilitating access to higher level of care or proceed with a particular treatment course
False information from test results	Initial positive results leading to need for further tests (or a sequence of tests) to further confirm/rule out a condition (false positives) False reassurance from test results (false negatives)
Incidental and indeterminate findings	Indeterminate or inconclusive results leading to further downstream testing (‘testing cascade’) in attempts to arrive at a definitive diagnosis Unexpected findings that may or may not have clinical significance, but can lead to additional testing

This table was created by the authors and no permission is required.

### Domain 2: physical effects of the test or testing process

Preparing for imaging tests was associated with specific unpleasant experiences, such as undergoing bowel preparation prior to colonography (table 2). However, more prominent outcomes were pain and physical discomfort while undergoing the imaging procedure (eg, mammography), as well as its immediate consequences. In some instances, the discomfort from an imaging modality depended on the area of the body being examined, for example, transvaginal versus abdominal ultrasound. Other physical outcomes included the effects of ionising radiation, which was mainly cited by patients undergoing frequent imaging.

**Table 2:** Patient-centred outcomes within domain of physical outcomes from imaging tests

Outcomes	Definition/explanation
Preparation for the test	Undergoing preparation such as bowel preparation, or fasting, or ensuring full bladder
Physical discomfort, tolerability during the test	Pain from interventions or manipulation needed as part of testing procedure Bruising from body parts being compressed or held in certain positions
Longer term physical effects	Reactions to contrast material Cumulative effects of ionising radiation

This table was created by the authors and no permission is required.

### Domain 3: emotional impact of the test or testing process

Tests used to evaluate new and/or concerning symptoms provided reassurance and relief when results ruled out certain conditions (table 3). Even in situations when results indicated an underlying condition, some patients reported feelings of relief that they had found the cause of their concerns. However, testing also produced anxiety and negative emotional outcomes that could occur before the test (anticipation anxiety), and afterwards while waiting for results, and as a result of test findings. During the imaging procedure, the physical constraints, confined spaces, noise and unfamiliarity of the procedures could lead to feelings of claustrophobia and distress, as well as embarrassment from having to undress. On some occasions, negative emotional outcomes resulted from disappointment or regret about initiating testing, from test results that did not answer concerns or revealed information that did not advance their care.

**Table 3:** Patient-centred outcomes within domain of emotional outcomes from imaging tests

Outcomes	Definition/explanation
Reassurance, relief	Relief or reassurance after finding symptoms not caused by serious condition
Anxiety, worry	Fear or anxiety waiting for testing to be performed and in anticipation of results Stress and anxiety while waiting to get test result Distress and other negative emotional impacts when test shows a serious condition, including false positive results
Claustrophobia, embarrassment	Claustrophobia, distress from imaging testing process (eg, narrowness of scanner, noise) Embarrassment or loss of modesty from exposing private body parts
Lack of control	Perceived lack of control over the test ordering, its conduct and the next steps the results lead to Feeling abandoned, isolated or helpless during the imaging test itself
Decisional regret, mismatch with expectations	Frustration or regret about uncertain test results leading to further testing Disappointment in test results that do not provide the information or findings expected, or that leave residual uncertainty

This table was created by the authors and no permission is required.

### Domain 4: test burden

Outcomes related to the direct or indirect burdens of imaging tests (table 4) included financial costs to patients, but these varied with the healthcare/health insurance system. These costs were particularly noted for complex imaging (MRI, CT, etc), and in situations where an initial, less costly, imaging test revealed findings that required more complex, and more expensive, subsequent imaging. Direct costs were not always expected or considered prior to the test itself, and few patients were aware of costs. Similarly, healthcare providers recognised the importance of cost, but lacked information about actual costs. Other test burdens included time off work and travel times particularly for patients living far from imaging centres. Occasionally, the results of imaging testing led to financial benefits to patients, for example, when imaging testing revealed conditions related to occupational or other injuries.

**Table 4:** Patient-centred outcomes within domain of burden from imaging tests

Outcomes	Definition/explanation
Financial costs of the test	Costs that the patient experiences directly or indirectly from the testing process itself Costs that arise from the clinical actions that the test leads to Potential financial benefits from workplace injuries or insurance or disability claims
Disruption to work or social life	Disruption to work, school, social activities from waiting for test to be performed, and undergoing the test itself, and waiting for test results (eg, time off work)

This table was created by the authors and no permission is required.

### Interactions between outcomes

In addition to the outcomes categorised within these four domains, PCOs interacted with, and influenced, outcomes in other domains. These interactions included, for example, knowledge or information provided by a test result (domain 1) influencing patients’ emotions (domain 3), or, a test that is more physically unpleasant (domain 2) that provides more valuable information (domain 1) than one that is less invasive. The pattern of interactions appeared to be complex and varied with factors such as test modality and clinical situation. Recognising these interactions exist suggests that weighing of risks and benefits across domains is likely to be challenging to incorporate into research or clinical care.

### Modifiers of outcomes

Multiple factors potentially impacted or modified whether or not a PCO occurred, its severity, its relative importance and its impact on the patient (table 5).^28^ These ranged from the characteristics of the individual patient, the type of test they are undergoing, the clinical situation, as well as the healthcare providers involved, the physical environment of the testing suite and communication of results. Embedded within several of these modifiers was the concept of a patient’s prior (pretest) probability of a particular outcome or condition. For example, a patient with a known history of a given condition may be at higher risk of recurrence (higher prior probability), whereas an asymptomatic individual being screened for that condition would be at a lower prior probability. These prior probabilities can influence outcomes following the test result (such as greater anxiety in the emotion domain).

**Table 5:** Factors that may modify patient-centred outcomes (PCOs) related to imaging tests

Modifier	Definition/examples
Individual patient characteristics	Sociocultural factors may impact response to multiple components of the testing process Prior experience of the test or testing process may influence knowledge and expectations of the test’s purpose and acceptance of related risks Prior probability of the condition being tested for, whether actual or perceived by the patient
Test type (ie, screening, diagnostic, monitoring)	Screening tests can lead to false positive results given large numbers of asymptomatic people being tested with relatively low prior probability Monitoring or surveillance for known condition (eg, cancer recurrence) can lead to anxiety in test intervals, and/or reassurance if results are negative
Clinical situation	Nature of the clinical condition being evaluated and its potential significance for that person’s healthcare may affect the balance of PCOs Testing in high acuity (eg, emergency department) settings may present different balance of benefits and risks (and prior probability of a given condition) compared with lower acuity (eg, primary care) setting
Clinicians and healthcare team	Perception of size and importance of test benefits and risks, based on experience, relationship with patient, healthcare setting, and knowledge or perception of an individual patient’s prior probability Ability (including time) to communicate indication for test is being used, relative risks and benefits Medical culture(s) may impose norms around test utilisation, acceptable levels of risk, patient expectations
Physical environment of imaging suite	Location in the clinic/hospital, visual appearance can influence patient emotions around the testing process Radiology staff can modify outcomes such as emotions and physical experiences through communication, trust and empathy
Communication of test results	Methods and timing of communicating results may impact the knowledge or information or emotional impact of the test

This table was created by the authors and no permission is required.

## Discussion

The Institute of Medicine highlighted that patient-centred practices are needed to address the psychological and social dimensions of patients’ healthcare concerns, in order to close quality gaps in healthcare.^39 40^ This study provides the first comprehensive attempt to define PCOs of imaging tests. We found that PCOs from imaging tests can be categorised into four domains: (1) information or knowledge yielded, (2) physical effects, (3) emotional outcomes and (4) test burden. We also noted that PCOs interact and influence each other in ways that are complex. Moreover, PCOs are not experienced identically; instead, they are influenced by factors related to the patient, the clinical environment and the physical environment of the test.

Our research advances an earlier concept that tests have more than ‘medical value’.^6 7 41 42^ While certain outcomes related to testing have been previously described, for example, emotional distress from false positive screening tests,^43–45^ a clear description and categorisation of PCOs related to imaging tests have largely been overlooked.^46^ ^47^

A strength of our recommendations is that they were informed by a wide body of primary and secondary research, and relied also on extensive input from a range of stakeholders, and are applicable to imaging testing generally, rather than one type of test (eg, screening) or a single imaging modality. We used consensus methods, following key steps that have been recommended for this type of process.^32^ Furthermore, a key area of guidance from our stakeholders was to focus on PCOs more directly related to the test and the testing process itself, rather than less direct (or indirect) impacts of the test on ‘downstream’ clinical management decisions and outcomes. We acknowledge there is limited literature to evaluate the validity, reliability and rigour of consensus methods,^32^ but we believe the methods used fulfil the criteria proposed by Hasson *et al*, namely credibility, applicability, auditability and confirmability.^48^ We used a process of prolonged engagement, with ongoing reflection of research findings both from research conducted by our team as well as wider literature, and based draft PCOs on evidence, and iterated on these over a period of 3 years including teleconference and a face-to-face conference with a heterogeneous group of stakeholders, representing patient, caregiver, clinician, researcher and industry perspectives. However, we acknowledge that there may now be value in further research to prioritise or rank the PCOs that we have described, using methods such as Delphi or nominal group technique.

The current focus on accuracy in evaluating imaging tests (and diagnostic tests in general) risks ignoring outcomes that may be meaningful from patients’ perspectives; it is the balance of outcomes that are important to patients, their caregivers and clinicians.^49^ However, patients and their clinicians currently lack information to make reasoned choices on the benefits and harms of diagnostic tests.^50^ Incorporating PCOs into diagnostic test evaluations has implications for guideline developers and policymakers. Measures of patient satisfaction typically focus on patient experience of services provided, overlooking PCOs. Fulfilling outcomes that are important to patients may lead to greater satisfaction. This can only be achieved for diagnostic testing if the outcomes that are important to patients are known and can be measured.^25 51 52^ Regulatory approval of new tests also focuses on test accuracy, although there is growing interest by the US Food and Drug Administration to consider additional impacts of tests in such decisions.

In order to advance the use of PCOs in research and implementation of imaging testing, we propose several next steps (box 1). First, validated measurement instruments exist for only some PCOs we identified, and few outcomes are collected using information from patients; there is a need to develop instruments to measure the range of outcomes we describe.^53^ This may be challenging when measuring the value of information (knowledge) from patients’ perspectives, and in considering the implications of the modifying factors we identified. Second, we need to determine the relative importance of outcomes to each other (and particularly to test accuracy). Evaluating risks and benefits of a given test is likely to vary with the severity, prominence or impact of the PCO related to that test. Balancing beneficial and harmful, as well as short-term and long-term impacts, may require more quantitative or discrete choice methods.^54^ Third, improved reporting of PCOs by extending current reporting standards for diagnostic accuracy studies^55^ could facilitate reporting of additional test outcomes.

Box 1 **Next steps needed to advance the use of patient-centred outcomes (PCOs) for imaging testing**

**Measurement**

- Validated measurement instruments for the full range of PCOs.
- Outcomes collected from patients themselves.

**Relative importance of outcomes**

- Methods to rank the importance of individual PCOs, and to balance positive (beneficial) and negative (harmful) outcomes, considering their severity, impact, timing of outcome.

**Reporting**

- Expanded standards for reporting PCOs in evaluation of imaging tests.

**Impacting care**

- Information on PCOs that can be collected and meaningfully shared with patients and the healthcare team to inform decision-making.

This box was created by the authors and no permission is required.

Finally, measuring PCOs is of little value if it fails to inform healthcare decision-making and quality of care.^51^ If the outcomes of imaging testing that are important to patients are known and can be measured, attempts to achieve these outcomes may lead to greater engagement with subsequent clinical management.^51 52^ Currently, however, for most tests and testing situations, patients, caregivers and providers lack information on PCOs. Efforts will be needed to collect information of PCOs and present this in ways that can be used to guide decision-making.^35 56^ We anticipate that this expansion of methods for test evaluation will stimulate new standards for research, reporting and use of PCOs, across the wider field of diagnostic testing beyond imaging tests.

## Data Availability

No data are available. We do not have data to share for this manuscript.
