# Supplementary material for: Patient-centred outcomes of imaging tests: recommendations for patients, clinicians and researchers
Source: BMJ Qual Saf. 2021 Oct 6;32(9):536–45. doi: 10.1136/bmjqs-2021-013311 (PMC10447372; doi:10.1136/bmjqs-2021-013311)
Supplement: Supplementary data [file bmjqs-2021-013311supp001.pdf]

Appendix 1: Methods and findings from scoping review of qualitative literature regarding patients' preferences in relation to imaging tests

Aim of the scoping review

Evaluation of imaging tests has been primarily concerned with demonstrating their ability to correctly 'rule in' or 'rule out' a diagnosis, and studies comparing test accuracy are used to guide regulatory approval and clinical adoption[1-4]. In order to describe more fully the range of patient-centered outcomes (PCOs) that are important to patients undergoing imaging studies we conducted a scoping review of qualitative research that described patients' experiences of imaging testing, to provide further evidence on the PCOs that had been identified by our primary qualitative research.[5 6] Our overarching research question was to describe adult patients' emotional, knowledge, and physical experiences and outcomes occurring before, during, or after undergoing imaging testing for any reason and any medical condition. We also sought to understand why these preferences were important to patients.

Methods used for scoping review

We followed Arksey and O'Malley's methodological framework for scoping reviews, supplemented by more recent recommendations and reported in line with the scoping review extension to PRISMA [7-9]. A protocol was developed and refined based on input from the Patient-centered Research for standards of Outcomes in Diagnostic testing (PROD) stakeholder group consisting of clinicians, methodological experts and patient representatives, convened to provide input to the PROD research program.

Search strategy

We developed a list of preliminary search terms related to the following overarching PCO themes identified from primary research conducted for the PROD study as well as additional literature on PCOs related to diagnostic tests: emotional reactions, physical effects, and the knowledge/information gained from imaging testing [6], this was facilitated by an Information Specialist and identification of keywords used in titles and abstracts of relevant articles known to the authors. Search terms were then mapped onto the relevant domains of the SPIDER framework for qualitative evidence synthesis (Sample, Phenomenon of Interest, Design, Evaluation, Research type) [10] to build a search strategy. The SPIDER tool was chosen as the domains allowed construction of an inclusive yet efficient search strategy. The search strategy was iteratively tested to determine the optimal search and the included terms and domains refined until agreement on the final strategy was reached between two authors (VH, MT). We searched a single bibliographic database searched (PubMed). Filters were applied for date range

(01/01/2003 through 06/28/2018), English language, and human subjects. (see terms available from authors).

Study selection

The final search results were imported into EndNote and duplicate articles removed. Titles and abstracts were screened for eligibility by one reviewer (VH); articles deemed potentially relevant were reviewed by a second reviewer (MT) and discussed for final determination of inclusion. Studies of original research conducted within the last 15 years were eligible for inclusion if they solicited patients' and/or caregivers' lived experiences of imaging testing. Caregivers' perspectives were included due to the integral role they have advocating, supporting and coordinating patients' healthcare. To capture narratives representative of the spectrum of patients undergoing imaging tests in routine practice, studies of patients with cognitive or physical disabilities were included. We limited study settings to high income countries (as defined by the OECD). We excluded neonatal, pediatric, or adolescent imaging studies, and interventional radiologic procedures to align with the PROD study aims. 'Snowballing' was used to identify additional studies from references of relevant full texts and any systematic reviews identified.

Data abstraction

An electronic data-abstraction spreadsheet was used to abstract data on author, country, population, setting, number of participants, imaging modality and purpose, and data collection methods/qualitative approach. One reviewer (VH) extracted the data of which 20% was checked by a second reviewer (MT). Qualitative data was charted against *a priori* PCO domains of 'emotion', 'knowledge' and 'physical'; excerpts consisted of participant quotes (with relevant participant descriptors where reported), and verbatim interpretation of the original data.

Collating, summarizing and reporting results

We used framework synthesis to synthesize results, which is appropriate when using a pre-existing 'framework' underpinned by previous research, and for conceptualizing the range of ideas (i.e. PCOs) being explored [11]. Data analysis was led by one reviewer (VH) and began by reviewing the included articles to become familiar with the findings. The same author re-read the articles in-depth, highlighting and making annotations against words, sentences and sections of text (participant quotes and authors' interpretation) that related to emotion, knowledge/information gain, and physical aspects of imaging testing. New PCOs within each of the pre-established domains were allowed to emerge from the coded data, forming subthemes. Themes were discussed with two reviewers (MZS, MT) and consensus

reached. During this process, we reflected on any impacts that our own preconceptions and interests could have had on data interpretation. To ensure balanced interpretation and presentation, findings were reviewed by stakeholders. The organization and analysis of qualitative data was facilitated by NVivo.

Results

Study selection

The search identified 5,472 articles, after removing duplicates, which were screened to identify 22 potentially includable articles. Following full text review, 19 qualified for inclusion in this review. A further 6 relevant articles were identified from snowballing, providing a total of 25 articles included in this review (**Figure**).

Figure. Flow of included studies

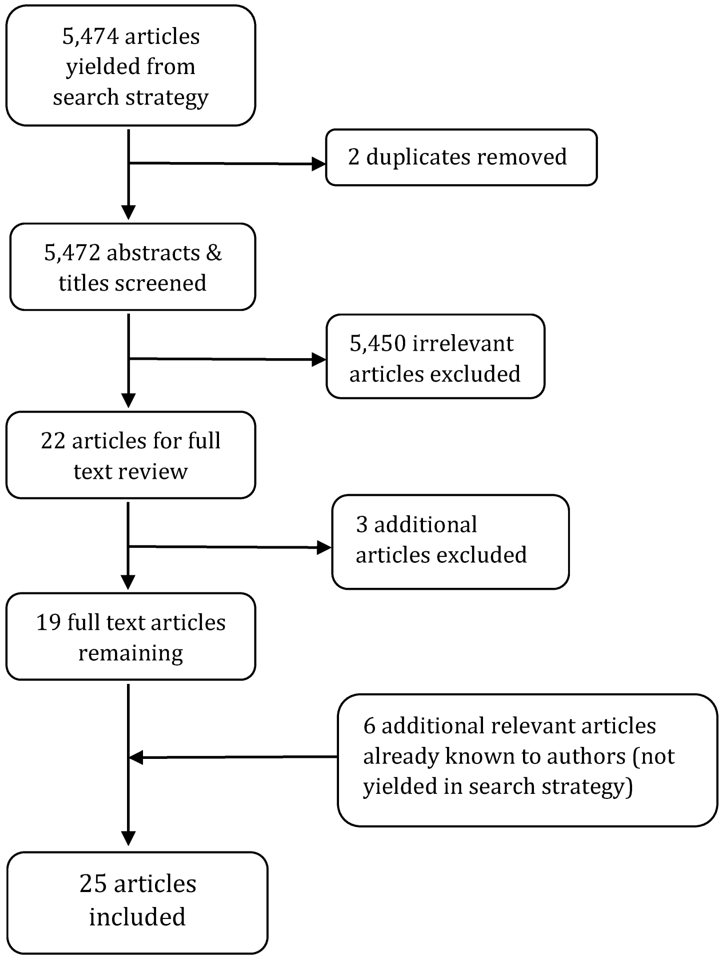

Note this figure was created by the authors and no permissions are required.

Characteristics of included studies

Data on a total of 656 patients and 23 caregivers were included in the 25 studies, with the overall sample being predominantly women (78%) (**Table 1**). Included articles were conducted in the United States (9), United Kingdom (8), Sweden (3), New Zealand (2), Australia (1), Germany (1), and Spain (1). Imaging tests included mammography (10), MRI (4), PET/(CT) (2), CT (2), SPECT-CT (1), CT colonography (1), CCTA (1), two were of multiple imaging modalities, and in one study the imaging test was not clearly reported. The target conditions and/or outcomes reported in the included studies were breast cancer (7), oropharynx/oral cancer (1), lung and/or colorectal cancer (3), inflammatory arthritis (1), coronary artery disease (1), multiple sclerosis (1), false-positive test results (2), incidental/indeterminate findings (3), Alzheimer's Disease (1), and was unclear or not reported (5). In studies where both patient and staff views are presented, only the patient views are extracted.

Table 1. Characteristics of Included Studies

Author, year (country)	Population	Gender; race/ethnic composition	Imaging modality	Purpose	Target condition / outcome	Data collection method
Andersson et al., 2017 (Sweden) [12]	Patients with confirmed head and neck cancer (n=9)	7 men, 2 female (aged 48-75 years); not reported	F-FDG PET/CT with fixation mask	Staging and radiation treatment planning	Oropharynx or oral cancer	Conversational interviews within 1 week of imaging
Bourke et al., 2017 (New Zealand)[13]	Patients with confirmed or suspected inflammatory arthritis with peripheral joint imaging test in preceding 6 weeks (n=33)	17 female, aged 25-83 years , 70% New Zealand, 12% New Zealand Maori	Conventional radiology, Ultrasound, MRI, CT	Diagnosis and management	Inflammatory arthritis (included rheumatoid, psoriatic, gout and undifferentiated inflammatory arthritis)	Semi-structured interviews within 6 weeks of imaging
Bond et al., 2015 (UK)[14]	Asymptomatic women who had experienced false-positive mammogram (n=21)	21 female aged 42-69 years; not reported	Mammogram	Screening	Breast cancer	Semi-structured interviews held between 0.5 and 12 years since false-positive mammogram
Brand et al. 2014 (Germany) [15]	Multiple Sclerosis patients with relapsing-remitting course (n=5)	All female aged 22-48 years; not reported	MRI	Management	Multiple Sclerosis	Semi-structured interviews
Carlsson et al. 2013 (Sweden)[16]	Patients undergoing variety of imaging examinations (e.g. brain, spine, pelvis, and hip) (n=10)	5 male, 5 female aged 21-70 years; not reported	MRI	Diagnosis	Unclear	Semi-structured interviews
Devcich et al. 2013 (New	Non-acute cardiac patients attending	Not reported (aged 39-71 years)	CCTA	Diagnosis	Coronary artery disease	Semi-structured interviews

Zealand)[17]	heart clinic (n=13)					conducted immediately following CCTA but prior to diagnosis, and immediately after communication of test results during cardiology consultation
Engelman et al., 2005 (USA)[18]	Women who underwent a mammogram within the previous 3 years with no history of cancer (n=103)	103 female aged 40-83 years; 53% Hispanic, 15% Black, 32% Non-Hispanic White	Mammogram	Screening	Not reported	Focus groups conducted in rural and urban communities
Engelman et a., 2012 (USA)[19]	Women with no prior history of breast cancer with mammogram during the 36 months prior to focus groups (n=88)	All female aged 40-82 years; 55% Hispanic, 45% Non-Hispanic White	Mammography	Screening	Breast cancer	Focus groups stratified by racial/ethnic groups: Hispanic and non-Hispanic white women
Evans et al., 2017 (UK) [20]	Patients participating in trials investigating value of WB-MRI for accelerating cancer treatment (n=51)	31 male, 20 female aged 40-89 years; not reported	Whole Body-MRI	Staging	Lung and colorectal cancer	Face to face and telephone interviews within 63 days of test
Grill et al., 2017 (USA)[21]	Patients (n=10) and caregivers (n=23) for whom option of amyloid imaging had been discussed	*Patients:* 4 female aged52-83 years; 8 white, 2 Latino *Caregivers:* 14 female aged 38-89 years; 19 white, 3 Latino	PET	Diagnosis	Alzheimer's Disease	Telephone interviews including open-ended questions with patients (and caregivers) who did and did not complete the scan
Hafeez et al., 2012 (UK)[22]	Patients referred for conventional colonoscopy invited to undergo MR colonography 2 hours prior (n=18)	11 male, 8 female median age of 40.5 years; not reported	MR Colonography	Diagnosis	Inflammatory bowel disease and suspected colon cancer	Semi-structured interviews
Lown et al. 2009 (USA)[23]	Women who had a diagnostic mammogram within previous 12 months (n=13)	All female with a mean age of 54 years; all white	Mammogram	Diagnosis	Breast cancer	Focus groups
Lumbreras et al. 2017 (Spain)[24]	Participants identified from a population survey with experience of imaging in previous 12months (n=20)	8 male, 12 female aged 18-90 years; not reported	X-ray<br>CT<br>MRI<br>Mammography<br>Ultrasound	Unclear	Unclear	Focus groups
Mathers et al.,	Women aged 42-63	All female; not	Mammography	Diagnosis	Breast cancer and	Semi-structured

2013 (UK)[25]	years with diagnosis of cancer or attending for further investigations of breast abnormalities previously detected (n=16)	reported			previously detected breast abnormalities	interviews first conducted 1 to 23 years after original diagnosis. Additional interviews conducted for those attending subsequent breast imaging
Nightingale et al., 2012 (UK)[26]	Cardiac patients attending cardiac imaging (n=22)	13 female, 9 male with a mean age of 63.9 years; not reported	SPECT-CT	Unclear	Unclear	Semi-structured interviews conducted before and after imaging on the day of SPECT-CT procedure
Poulos et al., 2005 (Australia)[27]	Women attending breast screening programs (n=12)	All female	Mammography	Screening	Breast cancer	Not reported
Slatore et al. 2013 (USA)[28]	Asymptomatic veterans with incidentally detected pulmonary nodules planning to obtain follow-up imaging (n=19)	18 male with a mean age of 66 years; 17 white	Unclear	Unclear	Incidental pulmonary nodules	Interviews conducted mean of 154 days after nodule detection
Sullivan et al. 2015 (USA)[29]	Veterans with an incidentally detected pulmonary nodule (n=17)	16 male with a mean age of 64 years; 14 white	CT	Surveillance	Incidental pulmonary nodules	Interviews conducted after first and second annual follow-up CT scan
Thomson et al. 2015 (USA)[30]	Women with confirmed false positive screening mammogram result with no personal history of cancer undergoing secondary imaging testing (n=40)	All female aged 40-68 years; 45% African American.	Mammogram	Screening	Breast cancer	Semi-structured interview
Tornqvist et al., 2006 (Sweden)[31]	Patients who did and did not complete different MRI scans (e.g. brain, spine, abdomen, wrist) because of varying levels of anxiety about the test (n=19)	12 female, 7 male aged 22-73 years; not reported	MRI	Unclear	Unclear	Conversational interviews with patients who did and did not complete the scan
Truesdale-Kennedy et al., 2010 (UK)[32]	Women with borderline to moderate intellectual disabilities undergoing breast screening in	All female aged 31-69 years	Mammography	Screening	Breast cancer	Focus groups using a semi-structured topic guide

	previous 12months (n=19)					
von Wagner et al., 2009[a] (UK)[33]	Symptomatic patients who had recently undergone CT colonography, barium enema or colonoscopy (n=49)	35 female, 14 male aged 57-92 years; not reported	CT Colonography	Diagnosis	Colorectal cancer	Semi-structured interviews conducted within 3 months of CT colonography, colonoscopy, or barium enema
Whelehan et al., 2016 (UK)[34]	Women with satisfactory and unsatisfactory experiences of breast screening programs (n=22)	All female (aged 28-56 years); 20 White British/Scottish, 1 African, 1 Afro-Caribbean	Mammography	Screening	Breast cancer	Semi-structured in-depth face-to-face or telephone interviews within 6 weeks of test (3 interviews were conducted >3 years after screen)
Wiener et al. 2012 (USA)[35]	Patients undergoing surveillance of an indeterminate nodule identified during workup of a pulmonary symptom or an incidental finding during workup of a non-pulmonary symptom (n=22)	86% female with a mean age of 60.7 years; 77% white, 18% black, 4.5% Hispanic	CT	Surveillance	Indeterminate pulmonary nodules	Focus groups
Wilkinson et al., 2011 (USA)[36]	Women with intellectual disabilities (n=27)	All female aged 27-69 years; 24 white, 3 black	Mammography	Screening	Breast cancer	Semi-structured interviews

Abbreviations: OP = outpatients, CT = computerized tomography, MRI= magnetic resonance imaging, SPECT-CT = single photon emission computed tomography, CCTA = coronary computed tomography angiography, PET = positron emission tomography, FDG-PET = fluorodeoxyglucose-positron emission tomography.

Note: This table was created by the authors and no permission is required

KNOWLEDGE OUTCOMES

Desire to know what is wrong

Knowing *what* is wrong was important to the majority of (symptomatic) patients undergoing MRI (e.g. for staging of lung and colorectal cancer), PET (e.g. for suspected Alzheimer's) and SPECT-CT (for conditions including multiple sclerosis and disc herniation), whereas knowing *if* something is wrong was important among patients under surveillance for incidental findings (e.g. pulmonary nodules) (**Table 2**).[12 13 15 20 21 26 29 31 35] Although patients, irrespective of imaging test, were fearful of a positive result such as tests showing recurrence or metastasis, most wanted a definitive diagnosis so they could either pursue the care needed to help them manage their condition, or make future social and health care plans.[12 21]

Patients and caregivers (sometimes with a mixed understanding of what a test could reliably rule-out),[35] viewed information yielded from imaging tests as a stepping stone towards obtaining a resolution.[16 20 21] Overall, this desire for knowledge was often reported to motivate patients to have an imaging test - only a small number were reported to decline (one example was the use of PET for Alzheimer's[21] because they felt test results (whether positive or negative) would have little impact on their overall management plan or prognosis.[21] Getting a definitive diagnosis brought relief to many symptomatic patients, irrespective of the results. On the other hand, feelings of surprise or shock were reported among a small number of symptomatic patients who had not considered a serious illness as a possibility, and anxiety in those with indeterminate results. Furthermore, those with incidental findings experienced anxiety from not knowing whether the finding (e.g. a lung nodule) was malignant, or might eventually become malignant.[35]

Desire to know what to expect

Patients ranged from feeling well informed about what they might expect while preparing for the test or during the test itself (WB-MRI, colorectal cancer),[13] to feeling insufficiently prepared.[20 36] Specifically, they wanted information about any potential sensory (e.g. what they might feel when injected with contrast material) or physical experiences of the test (e.g. narrowness of the imaging machine, noises they would hear, or discomfort they could expect to experience).[36] This information was typically desired ahead of the procedure to help them prepare for the experience,[20] or manage underlying anxieties, or to reconcile what they had learned from friends or relatives who had undergone the test and/or internet searches. During the procedure, patients highlighted the importance of repetition of the instructions so they knew what to expect at each step. This made patients feel more in control of their own emotions, and made the test feel less intimidating.[19]

Desire to know the possible harms of the test

Overall, patients' views were mixed regarding possible harms associated with imaging tests. Some were aware of the potential for adverse reactions from intravenous contrast material and concern about (accumulated) radiation exposure from (repeated) advanced imaging modalities (e.g. CT, MRI).[24] Patients generally believed risks had not been adequately explained[26] or where they had been, they struggled to understand what had been communicated to them due to use of medical jargon.[24 26] Consequently, they expressed desire for clear and concise information about possible harms. Although lack of knowledge concerned some patients who wanted to be informed about the risks of tests, notably

radiation exposure;[17 24 26] they believed the benefits typically outweighed the risks and were unconcerned or dismissed any possible harms and informational needs.[24]

Rapid feedback of results

A desire for rapid feedback of imaging results was prominent among the majority of patients who underwent a range of imaging studies (CT, MRI, MR colonography and mammogram). The anticipation of receiving potentially life-altering information was difficult to manage for many. They felt anxious and 'in limbo', reluctant to make important life decisions or plans in case of bad news when those plans might have to be abandoned. Most were symptomatic and were waiting for a diagnosis (of bowel disease, multiple sclerosis, breast cancer) and expressed dissatisfaction or distress with the length of time taken for results to be released or reported to them (between 3 days and 5 weeks, reported in one study).[25] A few patients believed the time it took to receive results was inversely proportional to the gravity of those results and so were reassured (falsely) by slower communication of results.[28] Patients experiencing an exacerbation of a known disease (of inflammatory bowel disease [22] were less anxious about waiting for results. However, for most patients alleviating anxiety over the possibility of cancer (or its recurrence), patients (symptomatic, without a diagnosis) wanted results issued on the same day as the test, regardless of whether findings were positive or negative.[16 20] Preferences for method of result communication varied; some preferred notification in person or over the telephone,[28] others were satisfied with written communication if it enabled results to be issued more promptly.

Table 2: Knowledge outcomes: themes and illustrative quotes

**Desire to know what is wrong**	*"But from a standpoint of managing her care and figuring out how best to take care of her with her symptoms, I feel like the scan was really positive in that it let me know she probably couldn't go home and live by herself again and that I would really need to take her care in a direction that none of us anticipated or could have predicted"* [21]<br><br>*"These loud noises really paled into significance because in my body now I've got a nasty little house guest, which has now stayed, not welcome, I'm going to get rid. And this is part of the mechanism to get rid […]. And these are the pictures that would help me get that done"* [12]
**Desire to know what to expect**	*"if you're informed and you know what to expect then it's not so scary"*[19]
**Desire to know the possible harms of the**	*"I always wonder how much radiation I am going to receive because I have never studied it and no-one has ever told me."*[24]

**test**	*"There's a lot of strange fears about radiation and it's gone crazy"*[13]<br><br>*"If the physician thinks I need the test to improve the management of my disease, I consider that the benefit/risk balance is in my favor"*[24]
**Faster feedback of results**	*"Waiting for the results is absolute hell"* [25]<br><br>*"No test results yet. I am just hoping they didn't find anything and there is another avenue that I might go down. I am dreading it might be cancer"* [20]

Note: This table was created by the authors and no permission is required

EMOTIONAL OUTCOMES

Desire for compassion and empathy from radiography staff

Patients identified that compassion and empathy from radiography staff, both before in the preparation and during imaging, as influencers of their experience of imaging, particularly when undergoing screening mammography.[27 34 36] Although some appreciated a depersonalized approach by staff as one way to mitigate embarrassment, others felt that a 'clinical' demeanor or perceived lack of interest by staff could be distressing.[25] Given anxiety about the (anticipated or previously experienced) discomfort of the mammogram, the possibility of a diagnosis of breast cancer diagnosis, and vulnerability from being physically exposed, patients wanted radiography staff to demonstrate sensitivity in the manner in which they gave verbal guidance and physical assistance (during breast positioning);[23 32 34] and cultural awareness of their modesty. Although patients infrequently reported terminating the procedure as a result of the demeanor of staff[20] their manner contributed to them feeling reluctant to participate in future screening programs[36] whereas compassion and empathy helped patients to endure any discomfort and pain associated with the imaging test.[34]

Desire for reassurance that they 'are not alone'

Patients wanted to know that they were not alone in what they viewed as the unfamiliar and lonely environment of some imaging modalities, and were reassured by physical presence and/or verbal interaction with staff [20 26]. This was frequently identified as important to patients during MRI, CT and PET imaging procedures, where feelings of isolation, abandonment and helplessness dominated.[12 20 26] Whilst physical proximity of staff typically provided patients with the most reassurance, communication through the intercom (and trust that staff would respond to calls for help) was also helpful in making patients feel secure during image acquisition.[16 20 26] The emotional comfort of knowing staff were close

by was sometimes enough to influence whether the procedure was prolonged or prematurely terminated.[26 31]

Table 3: Emotional outcomes: themes and illustrative quotes

**Desire for compassion and empathy from radiography staff**	*""It's like they're handling a lump of meat. Sort of throwing it on a slab and doing something to it. That how I think I would feel if I had large breasts"* [27]<br><br>*"I just felt she didn't seem interested, you were just another number. It was quite uncomfortable and she just kept saying 'If you don't stand right, you will have to come back again'"* [25]<br><br>*"She was very comforting the whole time. Very good she was. You feel a bit vulnerable"* [31]
**Desire for reassurance that they 'are not alone'**	*"Have they forgotten me? I can't take it…it's getting worse and worse"* [16]<br><br>*"[it] just made me feel a bit confident that you wasn't on your own, you know?"* [20]

Note: This table was created by the authors and no permission is required

PHYSICAL OUTCOMES

Comfort of the imaging environment

Patients' perceptions and experiences of the imaging environment for mammography, MRI, WB-MRI, and PET/CT varied widely from being acceptable to provoking negative feelings.[20 31] Prior to the test, factors such as the location of the imaging suite itself could provoke negative feelings (e.g. imaging suite location in the hospital basement perceived to be close to the mortuary; the observed narrowness of the MRI scanner which eliciting worry about claustrophobia (particularly among patients who had experienced this before),[20 31] and the *'sterile'*, '*mechanical'* or *'impersonal'* physicality of the imaging device (e.g. the breast plates of mammogram) which prompted anxiety as patients imagined their breasts being flattened, contributed to these perceptions.

During imaging patients likened the narrowness of the scanner as like being on a sunbed, in a space shuttle or entombed, and the loud hammering noises at unpredictable intervals during data acquisition sometimes posed a threat to patient's self-control.[16] This sometimes resulted in interruption, or, in occasional cases termination of the scan when patients were unable to control their emotional reactions.[20 31]

Not all patients experienced such negative feelings; some felt no threat to self-control and were able to control their reactions to the imaging environment and completed the test with little effort, although neither prior imaging test experience or social background consistently helped patients prepare or navigate their emotional responses to imaging.[16] Having music in the scan room,[16] comfortable ambient temperature[26] and a mirror in MRI scanners allowing patients to see out of the tunnel were identified as important for reducing anxiety during procedures, particularly amongst patients struggling with self-control.

Comfort of the imaging procedures

Most patients reported some degree of physical discomfort with mammography, SPECT-CT and CT colonography testing. The unpleasant experiences included: being put in awkward positions and breast compression (mammogram),[19 34] lying still for prolonged periods of time during data acquisition (WB-MRI, SPECT-CT),[12 22] use of gas/water enemas to distend the colon (CT colonography) leading to feelings of tenesmus[22 37] and the fixation mask to ensure correct head and neck positioning during scanning (PET/CT).

However, patients held mixed views with regards to the perceived severity of the discomfort, with some describing sensations as discomfort.[34 37] In contrast, others very clearly articulated pain [37] Regardless of the perceived intensity of the discomfort experienced, sensations were typically reported as transient. The level of discomfort was exacerbated for patients with pre-existing musculoskeletal problems.[20] Patients appreciated when staff paid attention to positioning them comfortably, and stimuli such as a TV to distract them,[20] suggesting this was important to help patients manage the discomfort.

Table 4: Physical outcomes: themes and illustrative quotes

**Comfort of the imaging environment**	*I'm not claustrophobic that (enclosed space) doesn't frighten me*" versus: *"when I saw the small tunnel I thought, shall I go in there, and then I felt panic."*[31]<br><br>"*That was one of the worst ones that I've had to go through with the noise…I felt like something was going to fall off and hit me."*[20]<br><br>*"It doesn't bother me. I've worked in pipes and tunnels and all sorts of places."*[20] and *"so – I've been lying under huge filters where you couldn't take a deep breath without feeling your chest against the wall, and that worked all right. But now, it was a feeling of panic."*[16]

**Comfort of the imaging procedures**	*"it's not a painful painful, but it's just tender"* (mammogram) [34] and *"I felt very bloated at one time and it was slightly painful. It was as comfortable as anything like that could be. I did experience some pain in the beginning"* [37]<br><br>*''Lying in same position for an extensive time period and not being able to move is very uncomfortable and in addition there is some weight on your back for the scan, which gets unbearable after approximately 20 min. There should be something in the room for distraction during scan, something like a TV even without the sound as you have to hear the breathing instruction as well.'* [20]

Note: This table was created by the authors and no permission is required

Summary of key findings from scoping review

Patient-centered outcomes identified

Based on the qualitative research identified in this scoping review across a wide range of imaging modalities, patient groups and clinical areas we identified multiple outcomes within domains we had identified a priori, namely the information or knowledge yielded by a test, the emotional impact of the test, and effects on physical symptoms patients may experience during or after the test. Outcomes within each of these domains were both positive (beneficial), and others negative (harmful). For example, knowing the result of an imaging test might provide explanation for symptoms a patient is experiencing, yet may also yield inconclusive or incorrect results. The emotional impact of a test might lead to peace of mind or reassurance, or, provoke more anxiety or distress. In contrast, the physical effects of the test, or preparing for a test were usually reported as negative effects, such as pain or discomfort from the procedure, or concern about longer term effects such as radiation exposure.

Factors identified that can influence test experience and outcomes

Findings from this review also provides evidence for multiple factors that could influence the test experience. These included moderating factors which are conditions that influence an outcome (its presence or absence), and mediating factors which explain how or why this relationship might exist.[38] For example, clinical staff frequently appeared to moderate several PCOs experienced by patients; this effect was mediated by their demeanor and communication about what patients would expect during the procedure. A further moderator appeared to be patients' level of self-activation from prior imaging test experience, or the level of preparedness; this mediated effects on outcomes such as anxiety or

anticipated level of pain. Finally, the physical design and location of the imaging testing suite acted as a moderator, for example through causing more distress and anxiety due to a tight or enclosed space, or the physical location of the imaging office in basement locations.

Strengths and weaknesses of the review method used

Consolidating findings from multiple primary studies allows deeper and more transferable insights about a phenomenon that is often not possible from a single study. This type of synthesis of qualitative research has gained popularity in recent years as an evidence-based method for informing patient-centered healthcare. We followed scoping review methods, including using two reviewers to identify eligible studies, snowballing techniques, and approaching the identification of themes. We feel that this technique was ideal, as it allowed a broader review of a wide body of literature using a rigorous approach.

A potential weakness of this review where we emphasized breadth over depth, is that we may have missed some relevant primary literature. The primary literature we identified focused heavily on the period of time during or shortly after testing, and we identified little research on longer term outcomes. Short term negative experiences during a procedure (e.g. pain) may become less important over time, although there is some evidence that at least some negative experiences (e.g., distress from false positive mammogram results) can lead to longer term anxiety and changes in screening behavior. In addition, we did not identify literature on the outcomes and experiences of patients who had declined testing. A further limitation is that we did not conduct independent abstraction of data or coding to check reliability, nor did we conduct critical appraisal of the primary studies identified (consistent with methods for scoping reviews). Qualitative research is an ideal method to identify outcomes of importance to patients, but does not allow us to weight or rank these outcomes, nor are we able to determine their overall importance to the patient, compared to other parts of their health care journey. We focused on adults (and excluded studies on pregnant women) and acknowledge that the experiences and outcomes of pregnant women, teenager and children might differ from those of adults. Limitations in the primary evidence that we identified for this scoping review may have overlooked PCOs related to certain imaging modalities, clinical situations, or patient populations, particularly where there have been fewer qualitative studies. This may limit the generalizability of our findings.

References cited in this appendix

1. Mathers SA, Chesson RA, Proctor JM, et al. The use of patient-centered outcome measures in radiology: a systematic review. *Acad Radiol* 2006;13(11):1394-404. doi: 10.1016/j.acra.2006.08.010 [published Online First: 2006/10/31]
2. Gazelle GS, Kessler L, Lee DW, et al. A framework for assessing the value of diagnostic imaging in the era of comparative effectiveness research. *Radiology* 2011;261(3):692-8. doi: 10.1148/radiol.11110155 [published Online First: 2011/11/19]
3. Ferrante di Ruffano L, Hyde CJ, McCaffery KJ, et al. Assessing the value of diagnostic tests: a framework for designing and evaluating trials. *BMJ* 2012;344:e686. doi: 10.1136/bmj.e686 [published Online First: 2012/02/23]
4. Lee DW, Neumann PJ, Rizzo JA. Understanding the medical and nonmedical value of diagnostic testing. *Value in health : the journal of the International Society for Pharmacoeconomics and Outcomes Research* 2010;13:310-14. doi: 10.1111/j.1524-4733.2009.00597.x [doi]
5. Zigman Suchsland ML, Hardy V, Zhang Y, et al. Provider Perspectives of Patient Experiences in Primary Care Imaging. *J Am Board Fam Med* 2019;32(3):392-97. doi: 10.3122/jabfm.2019.03.180288 [published Online First: 2019/05/10]
6. Zigman Suchsland ML, Witwer E, Truitt AR, et al. Patient-Centered Outcomes Related to Imaging Testing in US Primary Care. *J Am Coll Radiol* 2018 doi: 10.1016/j.jacr.2018.08.021 [published Online First: 2018/11/30]
7. Arksey H, O'Malley L. Scoping studies: towards a methodological framework. *International Journal of Social Research Methodology* 2005;8(1):19-32. doi: 10.1080/1364557032000119616
8. Levac D, Colquhoun H, O'Brien KK. Scoping studies: advancing the methodology. *Implement Sci* 2010;5:69. doi: 10.1186/1748-5908-5-69 [published Online First: 2010/09/22]
9. Colquhoun HL, Levac D, O'Brien KK, et al. Scoping reviews: time for clarity in definition, methods, and reporting. *J Clin Epidemiol* 2014;67(12):1291-4. doi: 10.1016/j.jclinepi.2014.03.013 [published Online First: 2014/07/19]
10. Cooke A, Smith D, Booth A. Beyond PICO: the SPIDER tool for qualitative evidence synthesis. *Qual Health Res* 2012;22(10):1435-43. doi: 10.1177/1049732312452938 [published Online First: 2012/07/26]
11. Barnett-Page E, Thomas J. Methods for the synthesis of qualitative research: a critical review. *BMC Med Res Methodol* 2009;9:59. doi: 10.1186/1471-2288-9-59 [published Online First: 2009/08/13]
12. Andersson C, Roing M, Tiblom Ehrsson Y, et al. It's a question of endurance - Patients with head and neck cancer experiences of (18)F-FDG PET/CT in a fixation mask. *Eur J Oncol Nurs* 2017;29:85-90. doi: 10.1016/j.ejon.2017.05.009 [published Online First: 2017/07/20]
13. Bourke S, Taylor WJ, Doyle AJ, et al. The patient experience of musculoskeletal imaging tests for investigation of inflammatory arthritis: a mixed-methods study. *Clin Rheumatol* 2017:1-8. doi: 10.1007/s10067-017-3760-0

14. Bond M, Garside R, Hyde C. A crisis of visibility: The psychological consequences of false-positive screening mammograms, an interview study. *Br J Health Psychol* 2015;20(4):792-806. doi: 10.1111/bjhp.12142 [published Online First: 2015/05/07]
15. Brand J, Köpke S, Kasper J, et al. Magnetic resonance imaging in multiple sclerosis--patients' experiences, information interests and responses to an education programme. *PLoS One* 2014;9(11):e113252. doi: 10.1371/journal.pone.0113252 [published Online First: 2014/11/22]
16. Carlsson S, Carlsson E. 'The situation and the uncertainty about the coming result scared me but interaction with the radiographers helped me through': a qualitative study on patients' experiences of magnetic resonance imaging examinations. *J Clin Nurs* 2013;22(21-22):3225-34. doi: 10.1111/jocn.12416 [published Online First: 2013/10/15]
17. Devcich DA, Ellis CJ, Waltham N, et al. Seeing what's happening on the inside: patients' views of the value of diagnostic cardiac computed tomography angiography. *Br J Health Psychol* 2014;19(4):810-22. doi: 10.1111/bjhp.12080 [published Online First: 2013/11/21]
18. Engelman KK, Cizik AM, Ellerbeck EF. Women's satisfaction with their mammography experience: results of a qualitative study. *Women Health* 2005;42(4):17-35. doi: 10.1300/j013v42n04_02 [published Online First: 2006/06/20]
19. Engelman KK, Cizik AM, Ellerbeck EF, et al. Perceptions of the screening mammography experience by Hispanic and non-Hispanic White women. *Womens Health Issues* 2012;22(4):e395-401. doi: 10.1016/j.whi.2012.04.006 [published Online First: 2012/06/05]
20. Evans R, Taylor S, Janes S, et al. Patient experience and perceived acceptability of whole-body magnetic resonance imaging for staging colorectal and lung cancer compared with current staging scans: A qualitative study. *BMJ Open* 2017;7:1-10. doi: 10.1136/bmjopen-2017-016391
21. Grill JD, Cox CG, Kremen S, et al. Patient and caregiver reactions to clinical amyloid imaging. *Alzheimers Dement* 2017;13(8):924-32. doi: 10.1016/j.jalz.2017.01.001 [published Online First: 2017/02/09]
22. Hafeez R, Wagner CV, Smith S, et al. Patient experiences of MR colonography and colonoscopy: a qualitative study. *Br J Radiol* 2012;85(1014):765-9. doi: 10.1259/bjr/36231529 [published Online First: 2011/10/20]
23. Lown BA, Roy E, Gorman P, et al. Women's and residents' experiences of communication in the diagnostic mammography suite. *Patient Educ Couns* 2009;77(3):328-37. doi: 10.1016/j.pec.2009.09.019 [published Online First: 2009/10/13]
24. Lumbreras B, Vilar J, Gonzalez-Alvarez I, et al. Avoiding fears and promoting shared decision-making: How should physicians inform patients about radiation exposure from imaging tests? *PLoS One* 2017;12(7):e0180592. doi: 10.1371/journal.pone.0180592 [published Online First: 2017/07/08]
25. Mathers SA, McKenzie GA, Robertson EM. Experience of women with a diagnosis of breast cancer attending for breast imaging. *Radiography* 2013;19(2):156-63. doi: 10.1016/j.radi.2012.11.004
26. Nightingale JM, Murphy FJ, Blakeley C. 'I thought it was just an x-ray': a qualitative investigation of patient experiences in cardiac SPECT-CT imaging. *Nucl Med Commun*

2012;33(3):246-54. doi: 10.1097/MNM.0b013e32834f90c6 [published Online First: 2011/12/22]

27. Poulos A, Llewellyn G. Mammography discomfort: a holistic perspective derived from women's experiences. *Radiography* 2005;11(1):17-25. doi: 10.1016/j.radi.2004.07.002
28. Slatore CG, Press N, Au DH, et al. What the heck is a "nodule"? A qualitative study of veterans with pulmonary nodules. *Ann Am Thorac Soc* 2013;10(4):330-5. doi: 10.1513/AnnalsATS.201304-080OC [published Online First: 2013/08/21]
29. Sullivan DR, Golden SE, Ganzini L, et al. 'I still don't know diddly': a longitudinal qualitative study of patients' knowledge and distress while undergoing evaluation of incidental pulmonary nodules. *NPJ Prim Care Respir Med* 2015;25:15028. doi: 10.1038/npjpcrm.2015.28 [published Online First: 2015/06/02]
30. Thomson MD, Siminoff LA. Perspectives on mammography after receipt of secondary screening owing to a false positive. *Womens Health Issues* 2015;25(2):128-33. doi: 10.1016/j.whi.2014.11.003 [published Online First: 2015/02/05]
31. Tornqvist E, Mansson A, Larsson EM, et al. It's like being in another world--patients' lived experience of magnetic resonance imaging. *J Clin Nurs* 2006;15(8):954-61. doi: 10.1111/j.1365-2702.2006.01499.x [published Online First: 2006/08/02]
32. Truesdale-Kennedy M, Taggart L, McIlfatrick S. Breast cancer knowledge among women with intellectual disabilities and their experiences of receiving breast mammography. *J Adv Nurs* 2011;67(6):1294-304. doi: 10.1111/j.1365-2648.2010.05595.x [published Online First: 2011/03/04]
33. Von Wagner C, Knight K, Halligan S, et al. Patient experiences of colonoscopy, barium enema and CT colonography: a qualitative study. *Br J Radiol* 2009;82(973):13-9. doi: 10.1259/bjr/61732956 [published Online First: 2008/10/01]
34. Whelehan P, Evans A, Ozakinci G. Client and practitioner perspectives on the screening mammography experience. *Eur J Cancer Care (Engl)* 2017;26(3) doi: 10.1111/ecc.12580 [published Online First: 2016/10/16]
35. Wiener RS, Gould MK, Woloshin S, et al. 'The thing is not knowing': patients' perspectives on surveillance of an indeterminate pulmonary nodule. *Health expectations : an international journal of public participation in health care and health policy* 2015;18(3):355-65. doi: 10.1111/hex.12036 [published Online First: 12/16]
36. Wilkinson JE, Deis CE, Bowen DJ, et al. 'It's easier said than done': perspectives on mammography from women with intellectual disabilities. *Ann Fam Med* 2011;9(2):142-7. doi: 10.1370/afm.1231 [published Online First: 2011/03/16]
37. Von Wagner C, Knight K, Halligan S, et al. Patient experiences of colonoscopy, barium enema and CT colonography: A qualitative study. *Br J Radiol* 2009;82:13-19. doi: 10.1259/bjr/61732956
38. Baron RM, Kenny DA. The moderator-mediator variable distinction in social psychological research: conceptual, strategic, and statistical considerations. *J Pers Soc Psychol* 1986;51(6):1173-82. [published Online First: 1986/12/01]

Appendix 2: Stakeholder List

Barbara Berney: (Patient & Consumer Advocate) – Co-Founder and President of the Vision Surgery Rehab Network, NFP

Becky Haines: (Radiology-American College of Radiology) – Senior Director, Press & Imaging 3.0, American College of Radiology

Bernhard Weigl: (Non-Governmental Organization) – Principal Investigator, Bill and Melinda Gates Foundation.

Beverly Green: (Methodologist) – Associate Investigator, Kaiser Permanente Washington Health Research Institute, Seattle.

Brian Do: (Patient & Consumer Advocate) – UW Undergraduate, Department of Human Centered Design and Engineering

Danielle Lavallee: (Health Services Researcher) – Research Associate Professor, Division of General Surgery, School of Medicine, University of Washington

David Kurth: (Radiology- American College of Radiology) - Senior Director, Practice Parameters and Appropriateness Criteria, American College of Radiology

Eunice Kim: (Student Researcher – Health Economics and Outcomes) – Doctor of Pharmacy student, University of Washington

Fiona Walter: (Methodologist) - Primary Care Cancer Research, Department of Public Health and Primary Care, School of Clinical Medicine, University of Cambridge, England

Gianna (Gigi) McMillan: (Patient & Consumer Advocate) – Graduate Program Coordinator, Bioethics Institute, Loyola Marymount University

Jerry Jarvik: (Radiologist/Health Services Researcher) – Radiologist, Adjunct Professor, Pharmacy and Orthopedics & Sports Medicine; Co-Director, Comparative Effectiveness, Cost and Outcomes Research Center

Karen Wernli: (Methodologist) – Associate Investigator, Kaiser Permanente Washington Health Research Institute, Seattle

Katherine Carter: (Radiology-Industry) – Regional Research Manager, West USA, GE Healthcare

Lisa Freeman: (Patient & Consumer Advocate) – Executive Director, Connecticut Center for Patient Safety

Matt Reilly: (Radiology-Industry) – Director, Clinical Research, Philips Healthcare

Matthew Thompson: (PROD PI/Primary Care/ Health Services Researcher) – Family Physician,; Helen D. Cohen Endowed Professor and Vice Chair for Research, Family Medicine, University of Washington

Monica Zigman Suchsland: (Research Scientist) – Family Medicine; Research and Sports Medicine Sections, School of Medicine, University of Washington

Pat Vigil: (Primary Care) –Family Physician, Central Washington Family Medicine Residency Program, Yakima, WA

Patty Spears: (Patient & Consumer Advocate) - Co-Chair of the Susan G. Komen Advocates in Science Steering Committee, and a Komen Scholar

Phil Posner: (Patient & Consumer Advocate) – Patient representative for the Food and Drug Administration, Department of Defense (CDMRP), PCORI Ambassador; Chair, WMATA Accessibility Advisory Committee; and volunteer with the National Capital MS Society

Robert Dubbs: (Patient & Consumer Advocate) – Retired, Healthcare Business, and Finance Attorney

Roger Chou: (Methodologist) – Professor, Medical Informatics and Clinical Epidemiology, School of Medicine, Oregon Health & Science University

Sally Lord: (Methodologist) – Head of Epidemiology and Medical Statistics, Notre Dame, and Senior Research Fellow of the National Health and Medical Research Council (NHMRC) Clinical Trials Centre in the University of Sydney, Australia

Steven Findlay: (Patient & Consumer Advocate) – Enterprising health policy analyst, advocate, communicator and writer/editor

Tom Trikalinos: (Methodologist) – Director, Center or Evidence-based Medicine (CEBM) at Brown University

Victoria Hardy: (Research Scientist) – Department of Family Medicine Research, School of Medicine, University of Washington

William Woodhouse: (Primary Care) – Family Physician and Clinical Professor, Department of Family Medicine, Idaho State University

Ying Zhang: (Primary Care) –Family Physician; and Assistant Professor, School of Medicine, University of Washington
